# Microbe biogeography tracks water masses in a dynamic oceanic frontal system

**DOI:** 10.1098/rsos.170033

**Published:** 2017-03-15

**Authors:** Anni Djurhuus, Philipp H. Boersch-Supan, Svein-Ole Mikalsen, Alex D. Rogers

**Affiliations:** 1 Department of Zoology, University of Oxford, South Parks Road, Oxford OX1 3PS, UK; 2 College of Marine Science, University of South Florida, 830 1st St SE, St Petersburg, FL 33701, USA; 3 Department of Integrative Biology, University of South Florida, 4202 E Fowler Avenue, Tampa, FL 33620, USA; 4 Department of Geography, University of Florida, Gainesville, FL 32611, USA; 5 Department of Science and Technology, University of the Faroe Islands, Noatun 3, Torshavn, Faroe Islands

**Keywords:** Southwest Indian Ridge, microbe biogeography, dynamic frontal systems

## Abstract

Dispersal limitation, not just environmental selection, plays an important role in microbial biogeography. The distance–decay relationship is thought to be weak in habitats where dispersal is high, such as in the pelagic environment, where ocean currents facilitate microbial dispersal. Most studies of microbial community composition to date have observed little geographical heterogeneity on a regional scale (100 km). We present a study of microbial communities across a dynamic frontal zone in the southwest Indian Ocean and investigate the spatial structure of the microbes with respect to the different water masses separated by these fronts. We collected 153 samples of free-living microorganisms from five seamounts located along a gradient from subtropical to subantarctic waters and across three depth layers: (i) the sub-surface chlorophyll maximum (approx. 40 m), (ii) the bottom of the euphotic zone (approx. 200 m), and (iii) the benthic boundary layer (300–2000 m). Diversity and abundance of microbial operational taxonomic units (OTUs) were assessed by amplification and sequencing of the 16S rRNA gene on an Illumina MiSeq platform. Multivariate analyses showed that microbial communities were structured more strongly by depth than by latitude, with similar phyla occurring within each depth stratum across seamounts. The deep layer was homogeneous across the entire survey area, corresponding to the spread of Antarctic intermediate water. However, within both the sub-surface layer and the intermediate depth stratum there was evidence for OTU turnover across fronts. The microbiome of these layers appears to be divided into three distinct biological regimes corresponding to the subantarctic surface water, the convergence zone and subtropical. We show that microbial biogeography across depth and latitudinal gradients is linked to the water masses the microbes persist in, resulting in regional patterns of microbial biogeography that correspond to the regional scale physical oceanography.

## 1. Introduction

The world's oceans are teeming with an enormous pool of diverse microscopic life forms. Ecologically, microbes play a vital role in marine food chains and global nutrient cycling and are involved in virtually all geochemical reactions occurring in the oceans [1,2]. A few studies have tried to tease apart depth and geographical distribution patterns of microbial taxa [3–7]. In the first global study of prokaryotic microbes by Pommier *et al.* [6], only two taxa, the Alphaproteobacterium, *Pelagibacter* *ubique*, and the photosynthetic cyanobacterial genus, *Synechococcus*, were found to be cosmopolitan. Furthermore, 69% of the identified operational taxonomic units (OTUs) were unique to their collection location. It has been demonstrated that species richness varies with season and peaks in high-latitude waters during winter [8–10]. Sul *et al.* [11] showed that most microbial OTUs did not exhibit a bipolar distribution and argued that their findings suggest that bacteria follow biogeographic patterns more typical of macroscopic organisms, and that dispersal limitation, not just environmental selection, probably plays an important role. The exact nature of the latitudinal gradients in richness, abundance and diversity in bacteria is still uncertain because of the substantial unexplained spatial and temporal variation of taxon occurrence; however, OTU richness has been shown to correlate with temperature, salinity, primary productivity and depth [5,12–15]. Changes in ocean currents and productivity may therefore be responsible for changes in observed bacterial and archaeal diversity. In addition, microbial community turnover has been observed across oceanic fronts in surface water masses [16], but less is known across water masses for deeper strata. The deep ocean is often considered a relatively uniform environment with stable physical parameters [4], with different microbial communities persisting in deep ocean water masses between ocean basins on a global scale.

The biogeography of microorganisms is undoubtedly directed by the evolutionary and ecological interaction of selection, genetic drift, dispersal and genetic mutation [17]. According to Hanson *et al.* [17], the distance–decay relationship, which states that the similarity between two locations declines as geographical distance increases, should be relatively weak in habitats where dispersal is high, such as in the pelagic environment, where ocean currents facilitate microbial dispersal. A distance effect on microbial community composition is most often observed at small (0–1 km) [18] or very large (more than 5000 *km*) spatial scales, i.e. between ocean basins [4,5]. A small-scale distance effect may be the result of microbial aggregation [19], which can be caused by dispersal limitation. Investigating microbial communities at regional scales (100 km) and across depth strata is imperative, as this is the scale at which different ocean masses create contrasting physical conditions and thus contrasting microbial communities [20]. Little is known about the depth distribution of microorganisms in many ocean basins, especially across mid-ocean ridges and the influence of those ridges on microbial dispersal. Open-ocean seamounts are considered to be ‘hotspots’ of marine life but their role in microbial dispersal is still under discussion [21,22]. They are often considered unique ecosystems in terms of their structure and sometimes high biomass of the benthic and pelagic biological communities [21,23–25]. While the ecology of metazoans on seamounts has received considerable attention, studies focusing on lower trophic levels, and microbial processes on seamounts in particular are lacking, despite their potential strong influence on biogeochemistry [26–29]. Seamounts are very dynamic hydrological habitats and may in some instances create local enhancement of large autotrophic cells and picoplankton (i.e. near the summit or flanks of seamounts) [27]. Surveying seamounts on the Southwest Indian Ridge (SWIR) we present a comparative study of the three-dimensional microbial biogeography around seamounts from the subantarctic to the subtropics in a dynamic frontal system.

This study focuses on differences in community composition on local (1–2 km) and regional (100 km) geographical scales, as well as along a 2 km depth range at each site. We aim to horizontally and vertically delineate the microbial communities of different water masses across one of the world's most hydrologically dynamic regions, the southwest Indian Ocean [30,31].

## 2. Material and methods

### 2.1 Sampling

**Figure 1.**
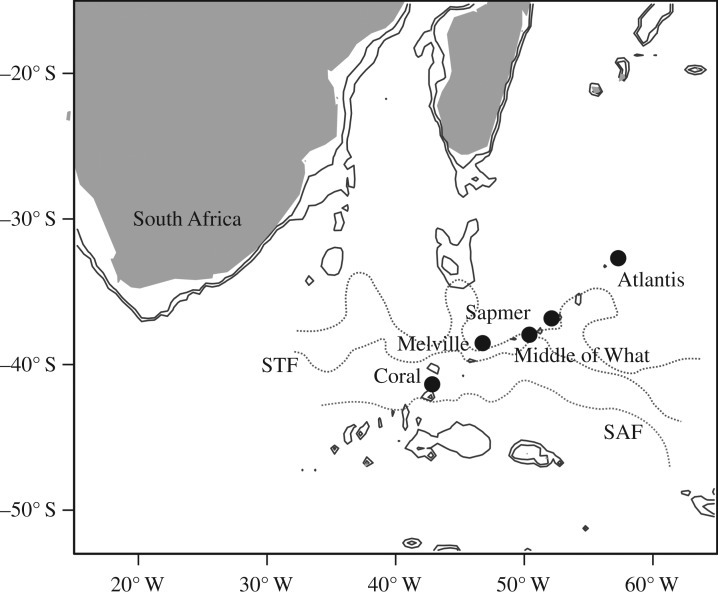
Locations of the five sampling stations on the Southwest Indian Ridge. The solid line represents the 1000 m contour. The dashed lines are the Agulhas Return Current, Sub-Tropical Front (STF), and Sub-Antarctic Front (SAF), from north to south [32].

Sampling was carried out during the RRS *James Cook* voyage JC66 from 4 November to 20 December 2011. Conductivity, temperature and depth (CTD) profiles, as well as all water samples, were collected with a SeaBird Electronics SBE +911 CTD and rosette fitted with Niskin bottles of 10 l volume. Samples were collected along transects across the seamounts with six CTD deployments on Coral, Melville, Middle of What and Atlantis seamounts and a single CTD deployment on the summit of Sapmer seamount (figure 1). An *in situ* fluorometer measured chlorophyll *a* fluorescence to a maximum depth of about 300 m [33] on all CTD deployments.

Seawater (1 l) was filtered using a 0.22 μ*m* filter from each CTD deployment at the chlorophyll maximum (40–80 m, referred to as shallow stratum), at the boundary of the euphotic zone (∼200 *m* depth, referred to as mid stratum), and 10–20 m (more than 500 *m* depth) from the seafloor (referred to as deep stratum). Two samples were collected from each depth layer from separate Niskin bottles. During the fieldwork, a total of 153 samples were collected for sequencing of microorganisms and 223 samples were collected for quantification of microorganisms through flow cytometry (table 1).

**Table 1.** Overview of samples collected at the five seamount locations. FC is flow cytometry samples. DNA, FC and Nutr. (nutrients) are number of samples. WM, water mass; SA, subantarctic; ST, subtropical; CZ, convergence zone.

station	longitude	latitude	summit (m)	WM	DNA	FC	Nutr.
Coral	42°50′31′′ *E*	41°21′23′′ *S*	198	SA	43	79	86
Melville	46°45′74′′ *E*	38°31′56′′ *S*	120	CZ	32	64	73
MoW	50°22′16′′ *E*	37°56′76′′ *S*	1078	CZ	32	74	81
Sapmer	52°07′24′′ *E*	36°49′63′′ *S*	446	CZ	11	12	53
Atlantis	57°17′26′′ *E*	32°42′01′′ *S*	713	ST	35	66	91

For every CTD deployment, water samples for nutrient analysis were collected at the same locations as particulate organic carbon and flow cytometry samples. Data from the flow cytometry and particulate organic carbon analysis were acquired from Djurhuus *et al.* [29]. We collected 173 samples for macronutrients, nitrite, nitrite+nitrate, phosphate and silicate. They were analysed using a five-channel Technicon AAII segmented flow analyser [34]. Analyses were calibrated, quality controlled, and checked against KANSO-certified nutrient reference materials. All environmental data were used in an analysis of drivers of microbial community structure; see multivariate regression analysis below.

### 2.2 Illumina MiSeq sequencing and preparation

DNA extraction, polymerase chain reaction (PCR) and sequencing were performed using a modified version of the protocol presented in Caporaso *et al.* [35], adapted for the Illumina MiSeq according to the Earth Microbiome Project (EMP) standards [36] (http://www.earthmicrobiome.org/emp-standard-protocols). In brief, the genomic DNA was extracted from sub-samples of the water filters using a Powersoil-htp 96 well DNA isolation kit (MoBio) with a 10 min (65°*C*) incubation step modification. The V4-V5 region of the 16S rRNA gene was amplified with 515F/806R primers with 12 base pair (bp) barcodes. Amplification primers were adapted from Caporaso *et al.* [36] to include nine extra bases in the adapter region of the forward amplification primer that support paired-end sequencing on the MiSeq. Amplifications were done in triplicate and followed the EMP PCR protocol. PCR products were pooled at equimolar concentrations and cleaned using the UltraClean PCR Clean-Up Kit (MoBio). 16S rRNA amplicon sequencing was conducted at the IGSB Next Generation Sequencing Core at Argonne National Laboratory using 151 bp paired-end sequencing on an Illumina MiSeq instrument.

Quality filtering of reads was applied, as described previously [36]. Reads shorter than 75 bases and chimeras and reads whose barcode did not match an expected barcode were discarded.

### 2.3 Bioinformatics

All bioinformatics were conducted using QIIME [37]. Forward and reverse raw sequences were combined using PEAR [38]. Joined reads were demultiplexed and quality trimmed. An open-reference OTU picking strategy was used, where OTUs were clustered against the GreenGenes 13_8 reference sequences using uclust [39] and reads with no hit to the reference sequence collection were subsequently clustered de novo at the 97% similarity level using uclust [39]. Reads were assigned to OTUs based on their best hit to this database at more than or equal to 97% sequence identity. PYNAST [35] was used to align OTU sequences and OTU taxonomy was assessed using the RDP classifier retrained towards the GreenGenes database (97% similarity) [40]. Median sequence counts per sample after OTU picking were 22 522 with a standard deviation of 8321. To generate a final OTU table, sequences not aligning in the PYNAST step were removed, and a sub-sampled OTU table was created by random sampling to an even depth of 11 270 sequences per sample and all singletons were removed.

Taxonomy was assigned to each read by accepting the GreenGenes taxonomy string of the best matching GreenGenes sequence. Data are available through Dryad at http://dx.doi.org/10.5061/dryad.qh767 [41].

### 2.4 Data analysis

R v. 3.2 (R Core Team, 2016) was used for all statistical analyses. For ordination and richness analyses, the R package vegan was used [42].

For species richness estimates, we used observed richness. Differences in bacterial abundances and richness between stations were compared using an ANOVA and *post hoc* Tukey HSD tests.

Variation of microbial community structure with depth was assessed using non-metric multidimensional scaling (NMDS) analysis on Bray–Curtis dissimilarities, with 10 000 random permutations.

Multivariate regression tree (MRT) analysis [43] was used to identify a hierarchy of environmental factors and their individual contribution to microbial community structure. This method performs hierarchical dichotomous clustering of community data by selecting environmental parameters that maximize the homogeneity within groups of samples. Accordingly, these clusters are characterized by both a homogeneous assemblage structure and similar covariate values. MRTs do not employ significance testing but use cross-validation to determine the optimal number of dichotomous splits and the importance of predictor variables [43]. We used the R package mvpart 1.6–0 [44] to perform the analyses on Bray–Curtis dissimiliarities [43] with salinity, temperature, depth, latitude, oxygen, particulate organic carbon, phosphate, silicate, nitrate and nitrite from previously published data [29]. Additionally, indicator species analysis [45–47] was performed using the R package indicpecies [45,47] to identify microbial taxa associated with splits of the MRT. This method aims at detecting OTUs that represent distinct ecological settings and indicate location-specific community types. This index is maximum when all individuals of a species are found in a single group of sites and when the species occurs in all sites of that group; it is a symmetric indicator.

Data were read into R, manipulated and visualized using the ggplot2 and phyloseq packages [48].

## 3. Results

### 3.1 Depth distribution of microbial communities

As shown in [29], the seamounts reflect the environmental setting in which they are situated. Coral had a relatively higher nutrient availability and lower temperature in the surface, reflecting the mesotrophic environment, while Atlantis had oligotrophic concentrations of nutrients, with a higher temperature in the surface layer. There were large differences in cell counts from the shallow strata between seamounts (electronic supplementary material, figure S1). Coral (the southernmost seamount with lowest temperature) had the highest average abundance (1.16×10^6^ cells ml^−1^±0.285) and Atlantis (northernmost seamount with highest temperature) the lowest (0.50×10^6^ cells ml^−1^±0.179) (Tukey HSD, d.f.=2, adjusted *p*<0.001), Melville and Middle of What had similar average abundances in the shallow layer (0.926 and 0.925×10^6^ cells ml^−1^±0.172, respectively) that were significantly different from Coral (Melville-Coral, Tukey HSD, d.f.=2, adjusted *p*=0.008, MoW-Coral, Tukey HSD, d.f.=2, adjusted *p*=0.006) and Atlantis seamounts (Melville-Atlantis, Tukey HSD, d.f.=2, adjusted *p*<0.001, MoW-Atlantis, Tukey HSD, d.f.=2, adjusted *p*<0.001).

On Coral, we observed a total of 27 544 OTUs, while Melville had 28 821 OTUs, Middle of What had 31 996 OTUs, and Atlantis had 21 988 OTUs similar to what was found in marine environments by Zinger *et al.* [49]. The observed OTU richness did not vary significantly between seamounts (ANOVA, *F*_3,49_=1.03, *p*=0.39; electronic supplementary material, figure S2).

**Figure 2.**
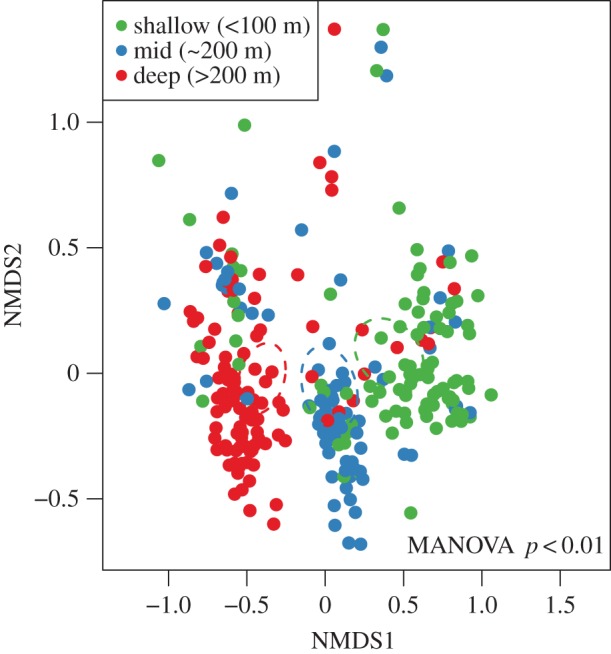
NMDS plot highlighting community differences between the three depth layers (shallow, 40–80 m; middle, ∼200m; deep, greater than 200 *m*). The ellipses represent the 99% confidence interval ellipses of the layer. MANOVA, multivariate analysis of variance; stress, 0.1225.

However, the middle stratum showed higher richness than the shallow and deep strata (ANOVA, *F*_3,92_=2.123, *p*=0.039, electronic supplementary material, figure S2; table 2). The strata also exhibited differences in their microbial community composition, demonstrated by the NMDS (figure 2; MANOVA *p*<0.01) with clear separation between the depth strata. Gammaproteobacteria dominated all depth layers, but the shallow layer had a higher abundance of the photoautotrophic class Synechococcophycideae compared with the middle and deep layers. In addition, the classes Flavobacteriia and Acidimicrobiia were more abundant in the shallow layer. Thaumarchaeota and Deltaproteobacteria both increased from shallow to deep (electronic supplementary material, figure S3).

**Table 2.** OTU richness, cell abundances and indicator species of the different strata of each seamount.

	station	observed richness	abundance (10^6^ cells ml^−1^)	indicator species	most abundant taxa	cluster (MRT)
shallow	Coral	1410	1.16 (±0.179)	Marine group III, SAR202	Synechococcaceae	6
	Melville	1486	0.926 (±0.172)	*Prochlorococcus*	*Prochlorococcus*	5
	MoW	1530	0.925 (±0.173)	*Prochlorococcus*	OCS155	5
	Atlantis	1421	0.50 (±0.179)	Pseudomonadaceae, Oceanospirillaceae	OCS155	2
mid	Coral	1134	0.297±0.067	*Nitrosopumilus*, HTCC	Oceanospirillaceae	1
	Melville	1780	0.311±0.095	*Synechococcus*, *Coraliomargarita*	Cenarchaeacea	4
	MoW	1805	0.266±0.118	*Synechococcus*, *Coraliomargarita*	Cenarchaeacea	4
	Atlantis	1502	0.249±0.0122	*Synechococcus*, *Coraliomargarita*	Cenarchaeacea	4
deep	Coral	1224	0.146±0.101	Halomonadaceae, *Candidatus* ‘portiera’	Oceanospirillales	3
	Melville	1433	0.129±0.105	Halomonadaceae, *Candidatus* ‘portiera’	SAR324	3
	MoW	1451	0.140±0.074	Halomonadaceae, *Candidatus* ‘portiera’	SAR324	3
	Atlantis	994	0.081±0.043	Halomonadaceae, *Candidatus* ‘portiera’	SAR324	3

Bacteria accounted for 86.5% and Archaea for 13.5% of all sequences in the entire study. The most abundant phylum was Proteobacteria (61.3%) with classes Gammaproteobacteria (26.6%), Alphaproteobacteria (16.8%) and Deltaproteobacteria (8.7%) being the most abundant. Other abundant classes were the Thaumarchaeota (9.7%), Acidimicrobiia (5.7%) and Synechococcaceae (3.5%; electronic supplementary material, figure S3). Thaumarchaeota accounted for 72% of all Archaea sequences. Two OTUs dominated in terms of abundance and accounted for approximately 10% of all sequences: one is classified in the family SAR324 (Deltaproteobacteria) and the other is from the order Oceanospirillales (Gammaproteobacteria). Both Oceanospirillales and SAR324 have a high abundance at all seamounts and all depths (electronic supplementary material, figure S3).

**Figure 3.**
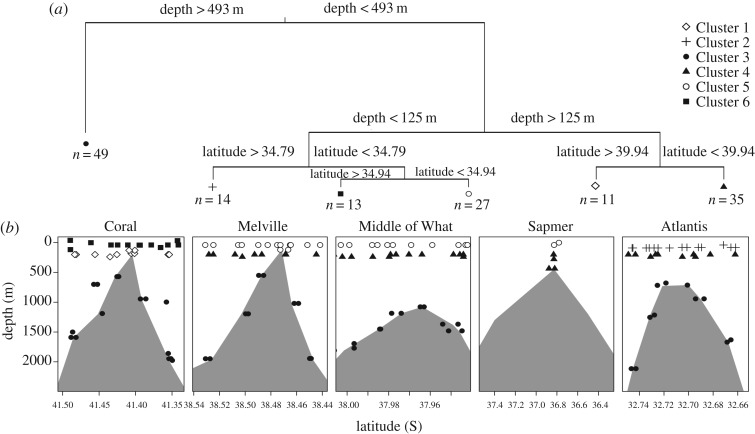
(*a*) Multivariate regression tree (MRT) of microbial communities and their structuring by latitude and depth. (*b*) Locations of MRT clusters across seamounts. Plotting symbols correspond to clusters in (*a*).

Optimal tree size for the multivariate regression analysis varied between 5 and 10 (electronic supplementary material, figure S4). An optimal tree size of 6 occurred most frequently (∼50% of all trees). Following Death *et al.* [43], we picked the tree of size 6 for further analyses, as it was within 1 s.e. of the optimal tree, and also the most frequent optimal tree. It revealed that depth and latitude together explained 66% of variation in community composition (figure 3). As indicated by the hierarchical order of splits and branch lengths of the MRT, depth was the main explanatory factor (split 1; figure 3), separating all seamount samples below 493 m (Cluster 3 in figure 3) from the rest. The cluster shallower than 493 m was again separated at 125 m, distinguishing the middle (Clusters 1, 4) and shallow layer (Clusters 2, 5, and 6). Coral seamount middle and shallow clusters are distinct from all other stations (Clusters 1, 6) and in the shallow layer (at the chlorophyll maximum) Atlantis clustered separately from all other stations (Cluster 2).

The indicator species analysis revealed that there were 286 taxa associated with the different clusters (table 2). From those 286, the two taxa most indicative of each cluster were as follows: chlorophyll maximum cluster of Coral was distinguished with Marine group III and SAR202 and the middle strata with the genera *Nitrosopumilus* (Cenarchaeaceae) and HTCC (Alteromonadales). The surface clusters of Melville, Middle of What and Sapmer were associated with the genus *Prochlorococcus* while the middle layer was associated with *Synechococcus*. The surface stratum of Atlantis was associated with *Pseudomonas* and Oceanospirillales and the middle was associated with *Synechococcus* and *Coraliomargarita*. All deep clusters were associated with *Candidatus* ‘portiera’ and Halomonadaceae.

### 3.2 Regional biogeography of seamount microorganisms

As demonstrated by the multivariate regression tree, depth is the main predictor of the microbial community structure across the survey area.

**Figure 4.**
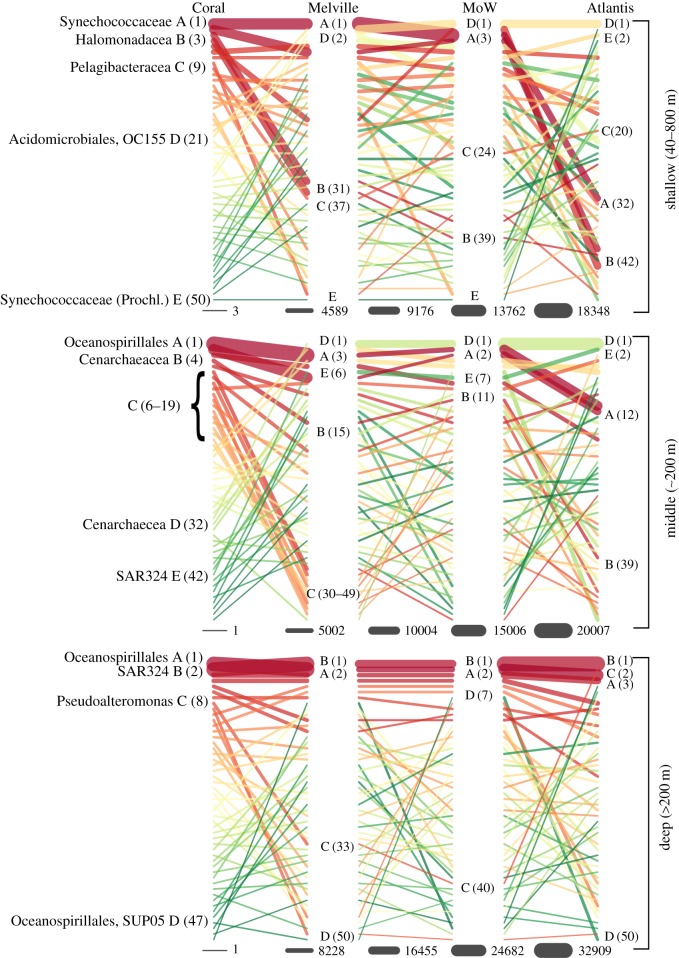
Rank abundance of the 50 most abundant OTUs on Coral, Melville, Middle of What and Atlantis. The rank abundance plots are divided between the three depth layers (shallow, 40–80 m; middle, ∼200 m; deep, greater than 200 *m*). Each line signifies the changes in a particular OTU and the colour of a specific line is to clarify the changes in rank across seamounts. Rank changes are indicated in parentheses on that specific seamount. Taxonomy of selected OTUs is specified on the left side of the figure. Line thickness represents relative abundance changes of a particular OTU as specified at the bottom of the figure.

The relative abundance of microbes between the seamounts differs at the class (electronic supplementary material, figure S3) and OTU levels (figure 4). Overall, the community structure, as captured by the rank abundance of OTUs, changes substantially between Coral and Melville and again between Middle of What and Atlantis. Melville and Middle of What are very similar across all individual strata (figure 4). There are large changes in rank abundances between the dominant OTUs from Coral to Atlantis. In the shallow layer the ranks of Synecoccocaceae (a) and Halomonadaceae decrease from dominating on Coral (ranks 1 and 3, respectively) to uncommon on Atlantis (ranks 32 and 42, respectively). The opposite latitudinal pattern, an increase in rank abundance, was observed for OCS155 and *Synechococcus* (Prochl.) from ranks 21 and 50 at Coral, respectively, to ranks 2 and 1, respectively, at Atlantis. In the middle layer, the changes in rank abundance are similar to those in the shallow layer, with the most abundant OTUs on Coral becoming the least abundant on Atlantis and vice versa. A large cluster of OTUs in the middle layer decreased in relative abundance from Coral to Melville (figure 4). These OTUs include Alteromonadales, HTCC2188 (OM182 clade), Flavobacteriaceae, Pelagibacteraceae, Marine Group II, Crenarchaeacea and Pseudoalteromonadaceae. The cluster occupies high ranks on Coral, but most of its OTUs are of lower rank on all other seamounts doing a taxa turn-over from Coral to Melville. The five most abundant OTUs in the deep layer are highly ranked throughout the seamounts. In general, the deep layer exhibits fewer rank changes of the dominant OTUs than the shallower strata, representing a more stable environment across the study area.

## 4. Discussion

### 4.1 Depth distribution of microbial communities

Our study reveals that the community composition of microorganisms along the SWIR is similar to open-ocean and deep-sea environments globally [5], with Alphaproteobacteria and Gammaproteobacteria being abundant throughout the water column. We found very similar relative abundances of all phyla to Sunagawa *et al.* [5], with the exception of much lower abundance of Alphaproteobacteria in our samples. In our study, the dominating primary producers are the Synechococcales, which have been found in similar abundances globally [29,50,51]. This is in contrast with Alves-Junior *et al.* [12] who found members from the order Prochlorales dominating at the surface and chlorophyll maximum layer in the southwest Atlantic Ocean at a similar latitude. In the shallow layer, we also found the actinobacterial clade, OCS155, which are heterotrophic and prolific producers of secondary compounds [52], probably relying on excretion of organic compounds from primary producers.

We found similar relative abundances of total Gammaproteobacteria in the deep layers to Alves-Junior *et al.* [12], although in our study it was dominated by the order Oceanospirillales, and not Alteromonadales. The MRT suggested distinct differences in community composition at depths greater than 493 *m* indicated by characteristic deep-sea microorganisms SAR324 and Oceanospirillales. SAR324 has been implicated in sulfur oxidation, carbon fixation and heterotrophy. The versatile metabolisms of SAR324 (lithotrophy, heterotrophy and alkane oxidation, all operating simultaneously) may explain SAR324's ubiquity in deep oceans [53]. Oceanospirillales is a psychropiezophilic microorganism, which explains its preference for the deep sea, and colder environments, such as the subantarctic Coral seamount rather than Atlantis, which is in subtropical waters [54]. Further, our samples collected at 200 m show higher abundance of Thaumarchaeota at depths just below the chlorophyll maximum, which is consistent with Sunagawa *et al.* [5].

Overall, we found that most of the abundant microorganisms in the deep layer were microorganisms with similar metabolic characteristics or environmental preference, enabling widespread geographical distribution across the deep layer. At the surface, we found a high abundance of microorganisms adapted to high light conditions, such as Synechococcaceae and the actinobacterial clade OCS155. The middle layer appears to be a mixture between the deep and shallow communities. It has a high abundance of several groups common in deep (e.g. Thaumarchaeota and Deltaproteobacteria) and shallow (Flavobacteriia, Alphaproteobacteria and Synechococcaceae). The layer below the chlorophyll maximum has been established as the area where most re-mineralization takes place in the water column, which may explain the presence of high abundances of heterotrophic microorganisms such as Pelagibacteraceae capable of degrading dimethylsulfoniopropionate released by decaying phytoplankton [55]. This might explain the pattern of higher species richness seen in the middle stratum (electronic supplementary material, figure S2) with a habitat structure similar to that of the deep sea but a stronger influence from the surface, creating an intermediate habitat where psychrophilic, piezophilic and primary producing microorganisms can coexist: some of them thriving and some existing as transient members, sinking away from the surface. The higher richness of the middle layer is enhanced in the convergence zone (Melville and Middle of What), creating a mixture of the deep-sea and surface microorganisms of both subtropical and subantarctic water masses.

It has been shown that primers used in this study can overestimate certain taxa, i.e. Thaumarchaeota and Gammaproteobacteria, and underestimate SAR11 in environmental samples [56,57]. This probably influenced the results of this study, especially in terms of relative abundance of the different phyla, causing the Gammaprotoebacteria to be dominating abundance and Alphaprotoebacteria less so (electronic supplementary material, figure S3). While we acknowledge the limitations of the primers used we do contend that any biases are consistent across the study, leaving the comparisons within the study unaffected. As all samples were sequenced using the same primer, they would all be biased in a similar fashion and the main conclusions of the study are not affected. The purpose of this study was to delineate patterns of community structures between locations and get an inference of what drives the microbial communities. We would argue that our patterns still hold true given that all the samples are analysed in the same way. Independent of primer, there will always be a bias towards or against certain taxa. It has been argued that reducing primer biases is especially important in the case of applications such as association networks or predicting functional processes [58], which is not the objective of this study.

For marine macrofauna, depth is a stronger predictor of metazoan community structure than geographical location [59]. Some studies have showed depth variation in microorganisms, but most focus has been on coastal areas or the surface layer of the open ocean [5,12]. Here we emphasize that, like macrofauna, the microbial community is segregated by depth [5,12,60]. Temperature has been argued to be the most important driver of depth changes; however, because temperature decreases with depth, its relative effects on microbial communities is difficult to disentangle [5]. Because temperatures decrease with depth, depth effect on microbial communities might not be caused by temperature but by the fact that the general physical environment changes markedly with water mass through depth [29]. Temperature is also an indicator of water mass, and can thus be further confounded with location when investigating biogeography on a basin or global scale. Depth appeared to be a stronger predictor of microbial community structure than geographical location, although we did observe geographical differences in the microbial communities of the euphotic zone at the northern and southern extremes of the survey area, when compared with the centre of the convergence zone.

Different seamount morphologies, as well as the variability of impinging currents, result in a broad range of hydrodynamic patterns, the relative strength and persistence of which may vary greatly in space and time [61]. Consequently, the effect of seamounts on biological communities may be highly intermittent and difficult to observe on the spatial and temporal scales accessible by vessel-based research. Mendonca *et al.* [27] observed higher microbial biomass and abundance on the summit of Seine and Sedlo seamounts in the North Atlantic Ocean, compared with a reference background sample. We cannot provide insight into potential differences between on-seamount and off-seamount samples, but we were able to investigate within seamount differences. All seamounts were relatively homogeneous within each depth layer and the MRT did not separate the summit or benthos of individual seamounts from the remainder of the samples. Although significant heterogeneity of microbial community composition has been described on local scales (1–10 km) [18], the depth division of our samples is greater than the between sample differences at the same depth at geographical distances of the order of 10–100 km. Metazoan community differences between the pelagos and the benthic boundary layer are well documented and have been observed on the SWIR [62], and elsewhere. However, little is known about differences between demersal and pelagic microbial communities. We did not observe marked differences between the microbial communities from samples with differing distances to seabed within a depth layer, although comparative samples are only available for Coral and Melville, given the deep summits of the other seamounts. It has been shown previously that the particulate organic carbon can be depleted on the summit of the same seamounts [29]; however, this was not reflected in differences in the community composition of samples from the summit versus the flanks. Given the sampling design of our study, distance to seabed is confounded with depth and so the study cannot unravel potential differences of the benthic boundary layer microbiome compared with open ocean microbial communities at similar depth. There is no difference between samples of the same depth relative to distance to seamount.

### 4.2 Regional biogeography of seamount microorganisms

The SWIR is located in an area where the Agulhas Return Current (ARC), the Sub-Tropical Front (STF) and the Sub-Antarctic Front (SAF), further to the south, create one of the most energetic and important hydrographic regions of the global ocean [63]. In the frontal zone (Melville, Middle of What and Sapmer), peak chlorophyll concentration in excess of 1 μ*g* *l*^−1^ has been recorded [63]. Outside this region, chlorophyll concentrations have been measured at less than 0.9 μ*g* *l*^−1^ [64]. Thus, seamounts along the SWIR are in contrasting productivity regimes and water masses depending on their proximity to the subtropical convergence zone and the SAF [29]. This trend is also reflected by the abundance of microorganisms, with higher abundances south of the SAF (Coral) and lowest abundances in the subtropical north (Atlantis) of the STF (Melville and Middle of What).

**Figure 5.**
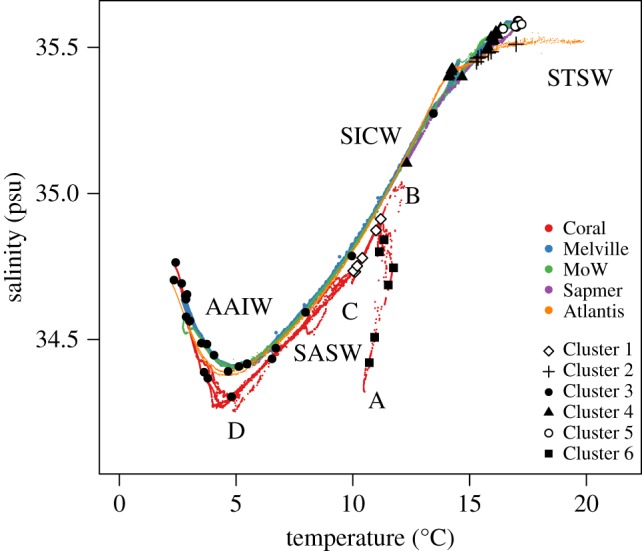
Temperature and salinity of water masses on SWIR seamounts. The points represent the clusters from the multivariate regression tree analysis. STSW is subtropical surface water, SICW is south Indian central water, SASW is colder subantarctic surface water, and AAIW is Antarctic intermediated water. The *t*/*s* properties showed a sub-surface salinity minimum (A). At the base of the fairly well-mixed surface layer, strong stratification marked the transition to a salinity maximum of 34.7–34.8 associated with a temperature maximum (B). At about 250 m an inflection in the *t*/*s* curve marked interleaving and small-scale minima and maxima in temperature and salinity (C). The segment C–B points to the end member of the STSW. C–D marks the area below the inflection point and points to the AAIW.

**Figure 6.**
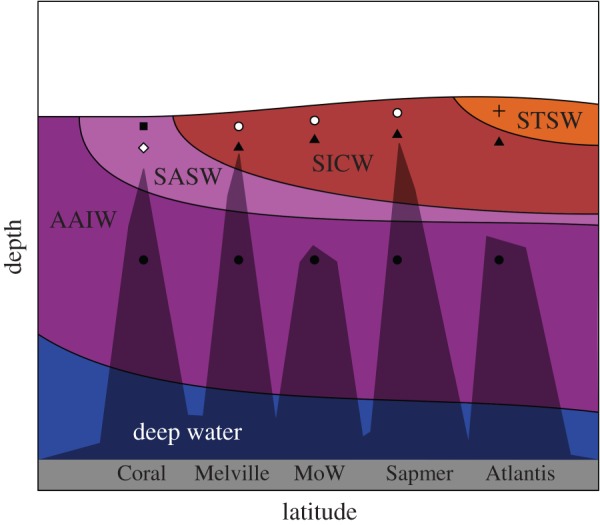
Schematic of water masses of the Southwest Indian Ridge according to Emery *et al.* and Tomczak *et al.* [65,66]. Multivariate regression tree clusters, representing different microbial community structure, are illustrated according to figures 5 and 3.

Microbial communities of corresponding depth layers in the north (Atlantis), convergence zone (Melville and Middle of What) and south (Coral) have similar abundant microorganisms at the order and phyla level, indicating adaptation to habitat rather than location for similar types of organisms. However, at an OTU level, the microbial communities show quite large differences between each location based on rank abundance and the MRT (figures 3 and 4). The MRT groups all deep seamount samples into one cluster, while separating out the surface and middle layer of Coral into two clusters, and the surface layer of Atlantis into another. As seen in the rank abundance, the surface layer on Coral is dominated by a *Synechococcus* OTU, which also dominates Melville and Middle of What, while Atlantis is dominated by *Prochlorococcus*. Both *Synechococcus* and *Prochlorococcus* have been shown in Djurhuus *et al.* [29] to be the dominant cyanobacteria at the surface of these seamounts. Interestingly, *Prochlorococcus* and *Synechococcus* are indicator species of the surface and middle layer, respectively, of the convergence zone seamounts distinguishing the different strata with their niche adaptations to high-light and low-light conditions. Photosynthetically available radiation is a major driver of primary producers, which will influence the surface communities, due to the strong environmental factor as seen on Coral, Melville, Middle of What and Sapmer (figures 5 and 6) [11].

In the middle layer, there is a group of OTUs that decreases from high rank abundance on Coral to low rank abundance on all other seamounts. The middle layer on Coral seamount was weakly stratified with maxima in temperature and salinity at 250 m depth [32]. The deep layer formed a single cluster signifying the stability of this environment with the deep Antarctic current dominating below 1000 m on all seamounts [67]. Atlantis was the seamount in the most strongly stratified waters [32] creating a very stable environment. As depth is the strongest predictor of microbial community structures, the stable stratified environment (i.e. no mixing between depth strata) on Atlantis would explain the largest difference seen in microbial community structure within the deep and shallow layers on Atlantis (table 2) between all samples, again indicating a depth effect that is stronger than a geographical effect.

The rank abundance plots further demonstrate the change in the microbial communities from south to north, through the convergence zone. The most abundant taxa on Coral are rare on Melville, with a similar shift between Middle of What and Atlantis, following the respective oceanic water masses and thus habitats (figure 6). This provides further evidence that water masses influence prokaryotic community composition and can be considered barriers to microbial dispersal [68]. Similar to that found by Agogue *et al.* [69], where they suggest the deep-water masses act as bio-oceanographic islands for bacterioplankton. In the deep sea, habitats have relatively little environmental variation (e.g. temperature, salinity), which has led to the evolution of species which have broad horizontal ranges [24]. However, because abiotic and biotic factors vary greatly with depth, many species possess restricted vertical ranges. Structuring of the microbial diversity is related to the physical, chemical and biological features of the water masses [68]; however, the definition of water masses by physical properties can be enhanced by the microbial ecology component as highlighted in this study (figures 5 and 6) [70–72].

The water mass separation based on temperature and salinity is in agreement with our grouping based on microbial diversity [73]. Based on the differences in communities that run from the microbial level up, the north (Atlantis), convergence zone (Melville, Sapmer, Middle of What) and south (Coral) could be considered three biogeographic zones. This is consistent with findings for macrofauna and megafauna, chemical and physical studies along the SWIR [29,62,73–75]. A mixture of environmental selection and dispersal limitation/facilitation probably plays roles in biogeographic patterns, although such a clear water mass separation has not been found previously in microbial communities [12]. However, the ability to detect a biogeographic pattern may depend on taxonomic resolution. As demonstrated in this study, the distance–decay relationship is clear between the most abundant taxa. Accordingly, the latter half of Baas–Becking principle ‘the environment selects’ combined with distance–decay, might be appropriate in this example where currents might facilitate adequate dispersal between the studied locations to continuously distribute microorganisms, but restriction of differing environments will compromise the success of the specific microbial taxa. However, the dispersal is not high enough to counteract the compositional differentiation imposed by the distance–decay relationship [17] and, contrary to ideas previously suggested by Hanson *et al.* [17], the distance–decay relationship is strong in the pelagic environment between seamounts in differing environmental setting (water masses), indicating an environmental selection. This demonstrates a regional biogeographic structure in the dominant microbial taxa, with semi-restricted dispersal by currents and very limited community mixing across water masses.

## Supplementary Material

Supplementary Material The supplementary information contains four figures S1-S4. These figures depict the abundance, richness, and phylum level taxa between the seamounts analysed in this manuscript. In addition, we have a supplementary figure graphically showing the error output from the Multivariate Regression Trees run on the microbial and environmental data.

## Data accessibility

All data are available through Dryad Digital Repository: http://dx.doi.org/10.5061/dryad.qh767 [41].
