# Supplementary material for: Microbe biogeography tracks water masses in a dynamic oceanic frontal system
Source: R Soc Open Sci. 2017 Mar 15;4(3):170033. doi: 10.1098/rsos.170033 (PMC5383857; doi:10.1098/rsos.170033)
Supplement: Supplementary Material The supplementary information contains four figures S1-S4. These figures depict the abundance, richness, and phylum level taxa between the seamounts analysed in this manuscript. In addition, we have a supplementary figure graphically showing the error output from the Multivari [file rsos170033supp1.pdf]

Supplementary Information for

Microbe biogeography tracks water-masses in a dynamic oceanic frontal system

Anni Djurhuus*, Philipp H. Boersch-Supan, Svein-Ole Mikalsen, Alex D. Rogers

*Corresponding author:
Email: anni.djurhuus@gmail.com

This PDF includes:
Fig S1 – S4

Online supplement

Methods

153 1L water samples were collected from the Southwest Indian Ridge for microbial analysis. The samples were sequenced on an Illumina MiSeq platform. The bioinformatics of the sequencing data was done in Qiime (Caporaso *et al.* 2011) and all statistical analyses and visualizations were done in the statistical software R (R Core Team, 2016). Data from the flow cytometry and POC analysis were acquired from Djurhuus *et al.* 2015.

For species richness estimates we used observed richness from the sequencing data. Differences in bacterial abundances and richness between stations were compared using an ANOVA and post-hoc Tukey HSD tests.

Multivariate Regression Tree (MRT) analysis (Death 2002) was used to identify a hierarchy of environmental factors and their individual contribution to microbial community structure. This method performs hierarchical dichotomous clustering of community data by selecting environmental parameters that maximize the homogeneity within groups of samples. Accordingly, these clusters are characterized by both a homogeneous assemblage structure and similar covariate values. MRTs do not employ significance testing but use cross-validation (CV) to determine the optimal number of dichotomous splits and the importance of predictor variables (Death 2002). We used the R package mvpart 1.6—0 to perform the analyses on Bray-Curtis dissimilarities with salinity, temperature, depth, latitude, oxygen, Particulate Organic Carbon, phosphate, silicate, nitrate, and nitrite from previously published data (Djurhuus *et al.* 2015).

Figures

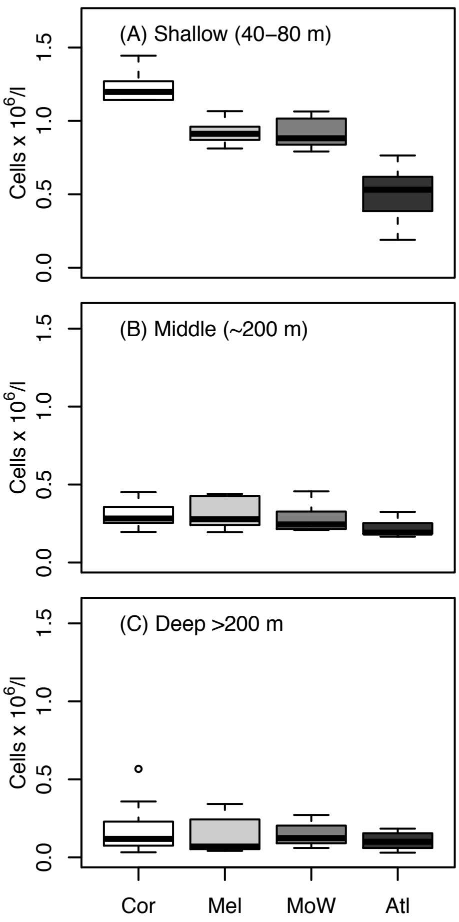

Figure S1
Abundance of microorganisms by depth stratum at the four seamounts, Coral (Cor), Meville (Mel), Middle of What (MoW), and Atlantis (Atl). A: shallow (40-80 m). B: mid (~200 m). C: deep (>200 m). Clearly visible abundance difference between Coral and the convergence zone (Melville and MoW) and between the convergence zone and Atlantis in the shallow.

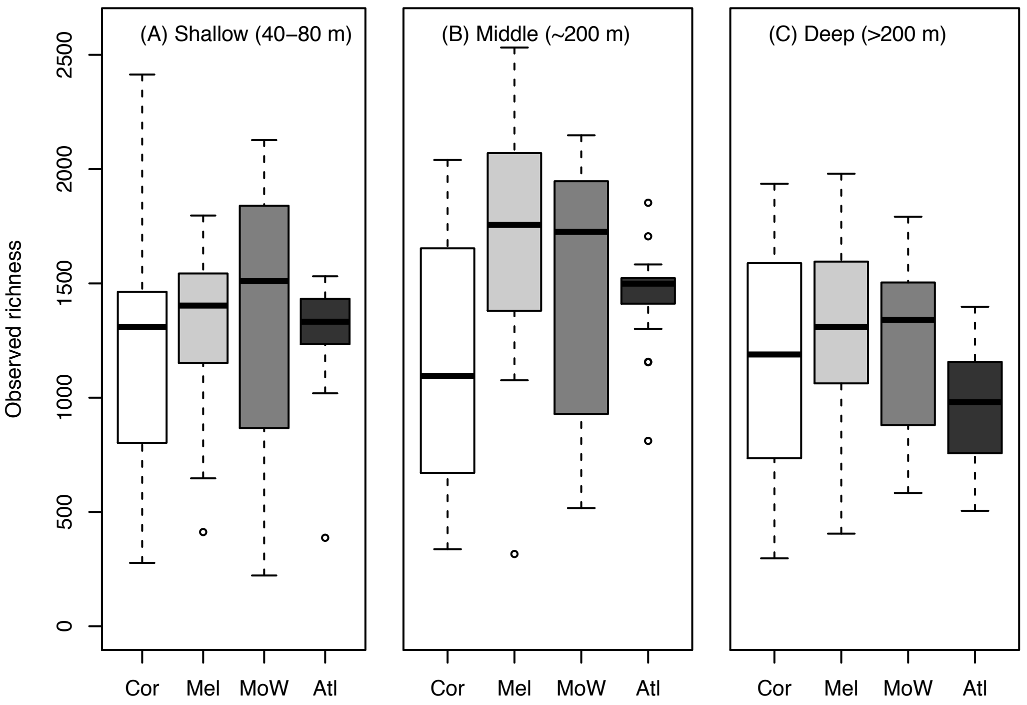

Figure S2
OTU richness across the four seamounts, Coral (Cor), Melville (Mel), Middle of What (MoW) and Atlantis (Atl). The richness plot is separated into the shallow (A, 40-80 m), middle (B, ~200 m) and deep (C, >200 m) stratum.

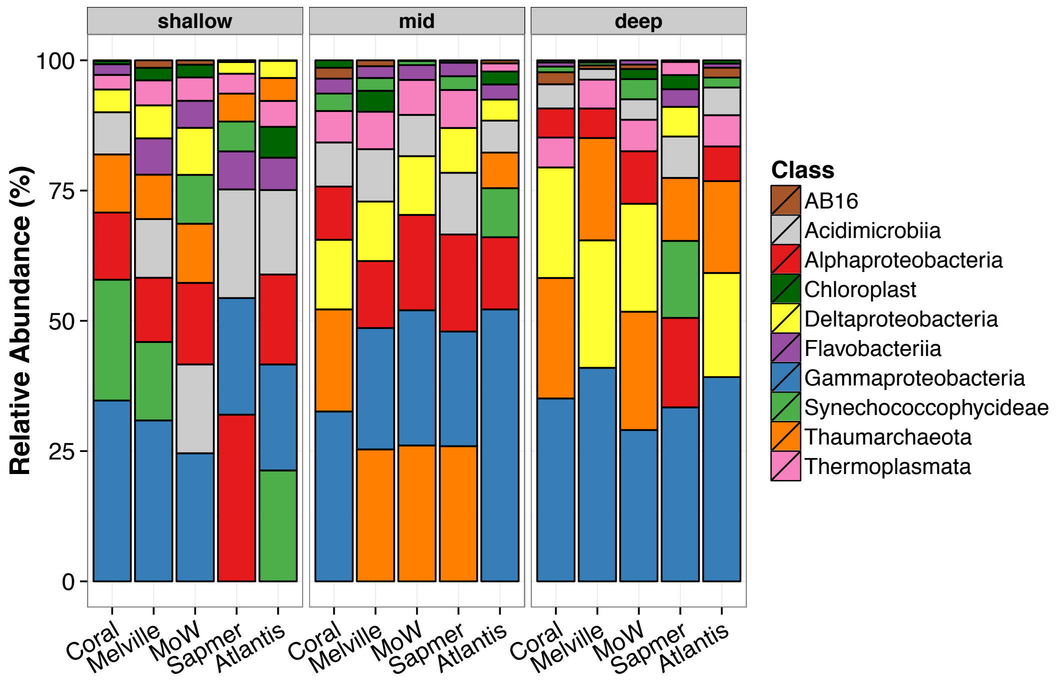

Figure S3
Barplot of the relatively most abundant microbial classes between depths (shallow=40-80 m, middle=~200 m, deep=>200 m) on all seamounts. MoW is Middle of What.

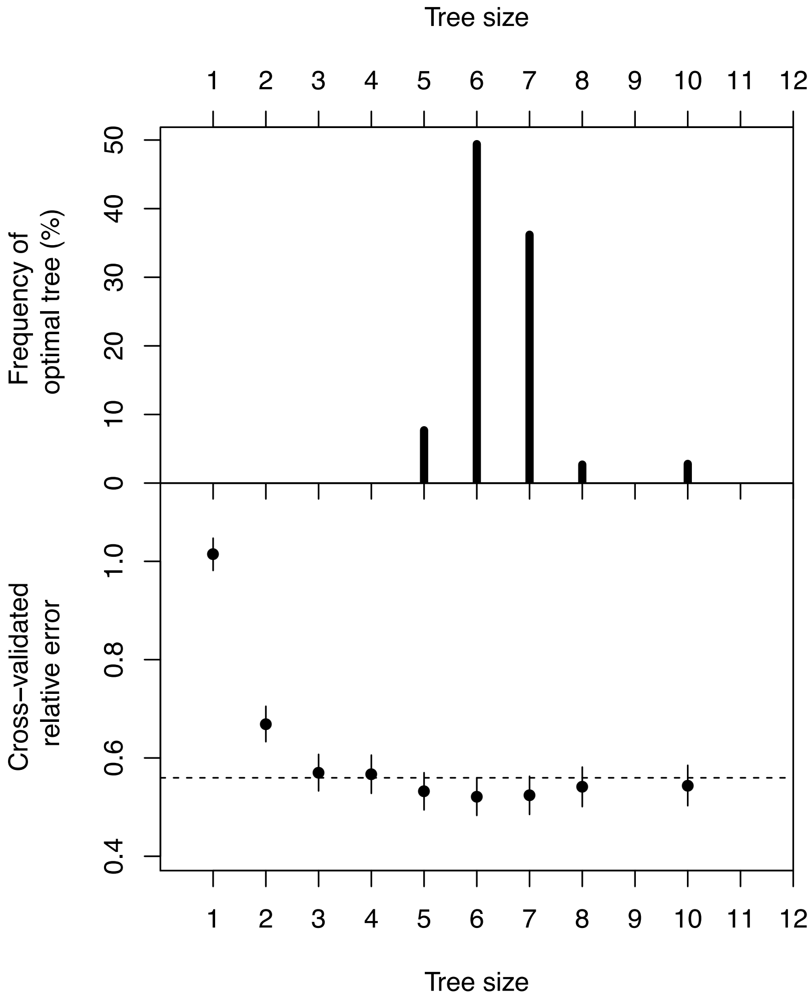

Figure S4
Multivariate Regression tree error output. The top plot is a histogram of how often the optimal tree is selected. The bottom graph displays the cross-validated error between the tree sizes. Optimal tree sizes were between 5-11. The most parsemoneous were tree sizes 5 and 9.

References

1. De'ath G, 2002 Multivariate regression trees: A new technique for mod eling species-environment relationships 83, 1105–1117. doi:10.1890/0012-9658(2002)083[1105:MRTANT]2.0.CO;2

2. Djurhuus A, Read JF, Rogers AD, 2015 The spatial distribution of particulate organic carbon and microorganisms on seamounts of the south west indian ridge. Deep Sea Research Part II: Topical Studies in Oceanography

3. Caporaso JG, et al., 2012 Ultra-high-throughput microbial community analysis on the Illumina HiSeq and MiSeq platforms. The ISME Journal 6, 1621–1624. doi:10.1038/ismej.2012.8
